# Single enzyme loaded nanoparticles for combinational ultrasound-guided focused ultrasound ablation and hypoxia-relieved chemotherapy

**DOI:** 10.7150/thno.37054

**Published:** 2019-10-17

**Authors:** Jianzhi Zhu, Zhicong Li, Changchang Zhang, Lizhou Lin, Shoupeng Cao, Hailong Che, Xiangyang Shi, Han Wang, Jan C. M. van Hest

**Affiliations:** 1 Bio-Organic Chemistry, Institute for Complex Molecular Systems, Eindhoven University of Technology, 5600 MB Eindhoven, the Netherlands.; 2 State Key Laboratory for Modification of Chemical Fiber and Polymer Materials, College of Chemistry, Chemical Engineering and Biotechnology, Donghua University, Shanghai 201620, People's Republic of China.; 3 Department of Radiology, Shanghai General Hospital, Shanghai Jiao Tong University School of Medicine, Shanghai 20080, People's Republic of China.; 4 Department of Ultrasound, Shanghai General Hospital, Shanghai Jiaotong University School of Medicine, Shanghai 20080, People's Republic of China.

**Keywords:** Catalase, polymer shell, pH-sensitive PEGylation, ultrasound-guided focused ultrasound ablation, chemotherapy.

## Abstract

Constructing nanosystems that synergistically combine therapeutic and diagnostic features is of great interest to the nanomedicine community but also remains a tremendous challenge.

**Methods:** In this work, we report novel catalytic nanoparticles composed of the enzyme catalase, encapsulated in a polymer shell and surface decorated with pH-sensitive poly(ethylene glycol) (PEGylated nCAT). These nanoparticles were used as a promoter for ultrasound (US)-guided focused ultrasound (FUS) ablation and hypoxia alleviation for application in Doxorubicin-based chemotherapy.

**Results:** The PEGylated nCAT produced highly effectively O_2_ from endogenous H_2_O_2_ to ameliorate the hypoxic and therefore poor-acoustic tumor environment. The generated O_2_ was utilized as 1) a contrast agent for US imaging; 2) strengthening agent for FUS ablation and 3) normoxia inducer to enhance chemotherapeutic efficacy. The PEGylated nCAT exhibited favorable enzyme activity after long-term storage, and after exposure to proteolytic conditions and elevated temperatures. The pH-responsive PEGylation contributed on the one hand to an extended *in vivo* circulation time over 48 h and on the other hand enabled PEG cleavage in the vicinity of cancer cells to facilitate cellular uptake.

**Conclusion:** The developed PEGylated nCAT can therefore effectively combine US-guided FUS and chemotherapy and can be regarded as a highly promising theranostic platform.

## Introduction

Focused ultrasound (FUS) is an innovative anti-tumor therapeutic technology, which has recently gained considerable research interest due to its non-invasive character, real-time capability, fast recovery and cost efficiency 1, 2. FUS is able to especially transfer the energy of the US to generate heat (>50 ^o^C) at the focused tumor site, thus resulting in tumor ablation 3. However, the current clinical FUS is still limited by the relatively low imaging resolution and poor heating generation speed, which lead to the disruption of healthy tissues along the irradiation pathway as a result of the prolonged treatment time. Therefore, gas bubble-based imaging and therapeutic agents have been developed to improve FUS therapy efficiency. In most cases, nano-carriers have been employed which under US pre-stimulation release gas bubbles that result in the amelioration of the acoustic environment inside the tumor 4, 5. Nevertheless, these gas bubble strategies suffer from exogenous US pre-stimulation and the lack of pre-imaging. Consequently, novel gas bubble strategies are needed for precise pre-imaging guidance and reduced irradiation time.

Owing to the high concentration of hydrogen peroxide (H_2_O_2_) inside the tumor cells, the catalytic generation of O_2_ bubbles from endogenous H_2_O_2_ holds great potential to achieve improved US imaging 6-11. To achieve this conversion, Wang *et al.* reported catalase (CAT) and superoxide dismutase encapsulated multifunctional hybrid nanogels; the dual enzyme-loaded hybrid nanogels efficiently enabled the catalytic reaction with reactive oxygen species (ROS) in the pathological environment to generate molecular O_2_, which changed the acoustic impedance of the tissue, thus enhancing US imaging 7. Liu *et al.* also developed on-demand H_2_O_2_-responsive CAT-loaded mesoporous organosilica nanoparticles as both contrast and synergistic agents for US-guided FUS cancer surgery 6. This hybrid nanoreactor could induce endogenous H_2_O_2_ decomposition to continuously release O_2_ bubbles for sustained contrast enhancement of US imaging and enlarged tumor ablation under FUS exposure. Therefore, this *in situ* generation of O_2_ bubbles using CAT from endogenous H_2_O_2_ not only omits the necessity of pre-irradiation for gas generation to enhance US pre-imaging for specific guidance of FUS surgery, but also ameliorates the poor acoustic environment of the tumor region to improve the FUS ablation. Nevertheless, this strategy is still limited by the high operational temperature accompanying the FUS process, which in turn causes the denaturation of CAT and limits further applications for cancer therapy.

Doxorubicin (DOX), as a traditional antineoplastic agent for chemotherapy, has been regarded to be effective in the treatment of cancer 12. The intracellular metabolite of DOX can react with O_2_ thus producing ROS, which ultimately results in the destruction of tumor cells 13. However, the effectiveness of DOX is considerably limited by the hypoxic tumor microenvironment, which is caused by the enhanced tumor proliferation and the abnormal vasculature 14, 15. Various efforts have been proposed to alleviate hypoxia to avoid the hypoxia-induced DOX resistance 16-18. For example, Zhang et al. developed a nanoplatform composed of MnO_2_ nanodots, which can catalyze endogenous H_2_O_2_ decomposition to generate O_2_; this relieves tumor hypoxia and improves the effect of DOX treatment 19. Importantly, Huang et al. fabricated calcium peroxide and CAT co-loaded alginate pellets which could significantly increase the chemotherapeutic effect of DOX by *in situ* generation of O_2_ to alleviate the hypoxic tumor region 16. Therefore, the aforementioned strategy to produce O_2_ bubbles from endogenous H_2_O_2_ for FUS surgery also holds great potential to overcome the hypoxia-induced drug resistance, thereby improving subsequent chemotherapy after FUS treatment.

**Scheme 1:**
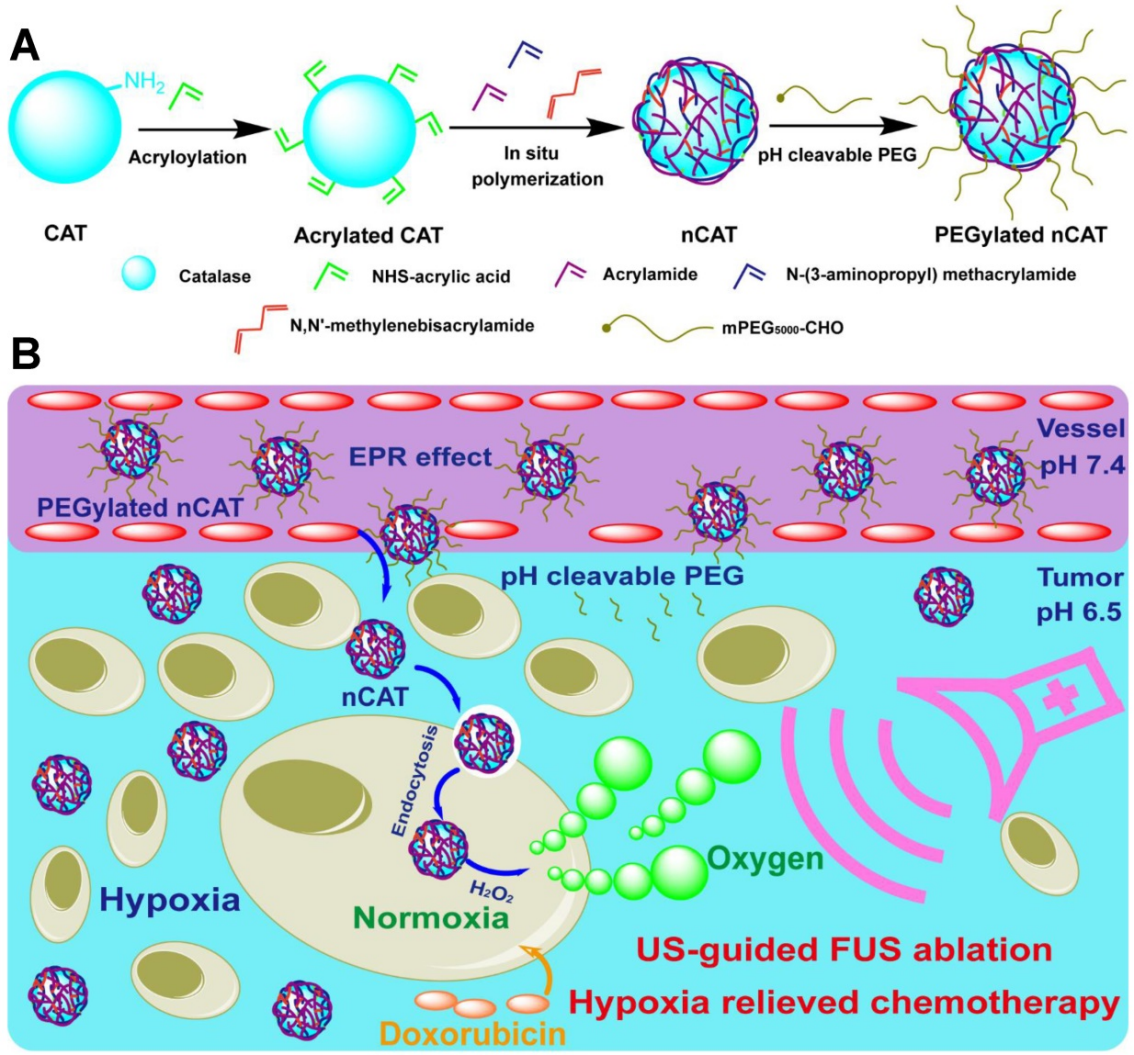
** (A)** Synthetic route for the preparation of PEGylated nCAT. **(B)** Schematic illustration of the use of PEGylated nCAT for combined US-guided FUS ablation and hypoxia-relieved chemotherapy.

In this report, we describe the development of tumor micro-environment responsive nanoparticles which are able to catalyze the conversion of endogenous H_2_O_2_ to generate O_2_ bubbles for US-guided FUS therapy and hypoxia-alleviated chemotherapy (Scheme 1). This nanosystem was facilely fabricated by *in situ* polymerization of acrylamide on the surface of CAT, followed by modification of the nanoparticles with pH-cleavable polyethylene glycol (PEG). When compared to previously reported nanoplatforms that encapsulate CAT via immobilization 6, 9-11 or electrostatic absorption 7 for US and/or FUS, the here developed nanoparticles provided CAT protection against enzyme dissociation even under the high operational temperature during the FUS process, ensuring that the enzyme retained its activity. More importantly, the surface modification with pH-responsive PEG significantly improved the *in vivo* circulation time whereas it bypassed the PEGylation-induced decreased cellular uptake. Finally, the hypoxic tumor microenvironment could be relieved after the nanoparticle-mediated FUS treatment, which therefore improved the subsequent conventional DOX chemotherapy. The carefully designed nanoparticles were thus able to serve as a tumor microenvironment responsive contrast and synergistic agent for US-guided FUS ablation and hypoxia alleviator for chemotherapy.

## Results and Discussion

### Synthesis and characterization of PEGylated nCAT

The PEGylated CAT nanoparticles (nCAT) were synthesized according to previous reports 20-22. As illustrated in Scheme 1A, native CAT was first conjugated with acryloyl groups via a reaction between the surface lysine groups of CAT and N-acryloxysuccinimide, followed by *in situ* polymerization using acrylamide and positively charged N-(3-aminopropyl) methacrylamide hydrochloride as monomers, N, N'-methylenebisacrylamide as crosslinker and ammonium persulfate and N, N, N', N'-tetramethylethylenediamine as initiators. The nCAT were further modified with pH-cleavable PEG via a reaction between the amine groups of the surface shell and PEG, chain-end modified with an aldehyde group to give PEGylated nCAT via reversible imine linkages. Bovine serum albumin (BSA) encapsulated nanoparticles (PEGylated nBSA) were synthesized as control following the same protocol.

**Figure 1:**
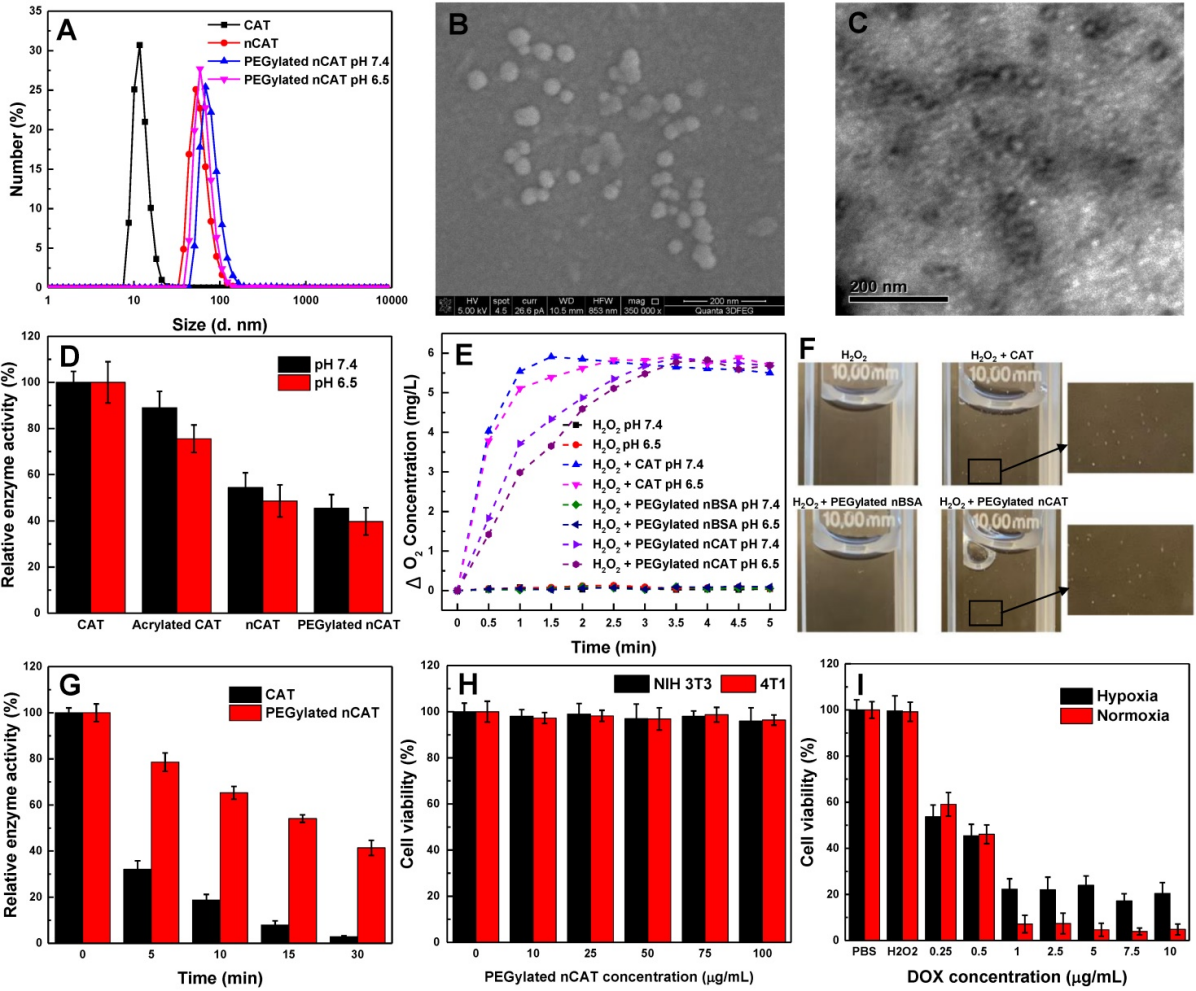
** (A)** Hydrodynamic size distribution of native CAT, nCAT and PEGylated nCAT at two different pH values. **(B)** SEM image of PEGylated nCAT. **(C)** TEM image of PEGylated nCAT stained with phosphotungstic acid. **(D)** Relative enzyme activity of native CAT, acrylated CAT, nCAT and PEGylated nCAT at pH 7.4 and 6.5. **(E)** Oxygen generation in H_2_O_2_ solutions (500 µM) incubated with native CAT, PEGylated nBSA and PEGylated nCAT at pH 7.4 and 6.5. **(F)** Photographs of O_2_ bubble generation in H_2_O_2_ solutions treated with native CAT, PEGylated nBSA and PEGylated nCAT. **(G)** Relative enzyme activity of native and PEGylated nCAT at different time points after incubation at 50 ^o^C. **(H)** Cell viabilities of NIH 3T3 and 4T1 cells treated with PEGylated nCAT at different concentrations for 24 h using CCK-8 assays. **(I)** Cell viabilities of 4T1 cells incubated with DOX at different concentrations under hypoxic and normoxic culture conditions for 24 h using CCK-8 assays.

The acryloylation of the surface of CAT was first confirmed by a fluorescamine assay, which indicated that on average 68 acryloyl groups were conjugated to one CAT (Figure S1). The synthesis of aldehyde-terminated PEG was confirmed using ^1^H NMR spectroscopy (Figure S2A). The fabrication of PEGylated nCAT was validated by dynamic light scattering (DLS), scanning electron microscopy (SEM) and transmission electron microscopy (TEM). As can be seen in Figure 1A, native CAT and nCAT displayed a hydrodynamic size of 11.7 nm and 52.7 nm, respectively, indicating the successful formation of the surface polymer shell. The hydrodynamic size of PEGylated nCAT at a physiological pH of 7.4 and tumor microenvironment pH of 6.5 was determined to be 68.1 nm and 58.8 nm, respectively, demonstrating the conjugation of pH-cleavable PEG and the corresponding pH responsiveness. The pH responsiveness was further confirmed using ^1^HNMR to identify the cleaved PEG (Figure S2B). The hydrodynamic size of PEGylated nCAT in phosphate buffered saline (PBS) and cell culture medium containing 10% fetal bovine serum (FBS) did not show an apparent change over the period of 14 d, suggesting that the PEGylated nCAT possessed favorable stability (Figure S3). Because CAT is shielded by the polymer shell, the zeta potential of PEGylated nCAT increased to -5 mV from -21.5 mV for the native CAT, which further confirmed the formation of nanoparticles. According to the representative SEM image in Figure 1B, PEGylated nCAT displayed a spherical morphology with an average diameter of ≈ 33 nm, which was also consistently observed in the TEM image (Figure 1C). This image also showed that the CAT core (transparent) was surrounded by a dark contrasting shell since the polymer could be selectively stained by phosphotungstic acid. Considering a polymer shell thickness of ≈ 11 nm (measured from the TEM image), the CAT core size was calculated to be ≈ 11 nm, which is comparable with the native CAT size of 9.0 nm × 6.0 nm × 2.0 nm 23, illustrating that only one CAT was encapsulated within the PEGylated nCAT. The DLS, SEM and TEM results therefore demonstrate that CAT was successfully enclosed into the PEGylated polymer shell.

### Enzyme activity of PEGylated nCAT

As a result of steric hindrance and mass transport resistance, a decreased enzyme activity tends to be observed when enzymes are encapsulated or loaded. Therefore, the catalytic activity of PEGylated nCAT was first investigated by recording the UV absorbance of H_2_O_2_ during CAT-mediated decomposition. As can be seen in Figure 1D, the enzyme activity displayed a decreasing trend during the modification process, and the PEGylated nCAT retained 45% and 39% of its initial catalytic activity at physiological pH of 7.4 and tumor acidic pH of 6.5, rspectively. The catalytic activity of PEGylated nCAT is however sufficient to mediate the decomposition of H_2_O_2_, which was further confirmed by measuring the O_2_ concentration change after co-incubation of PEGylated nCAT with a H_2_O_2_ solution. As shown in Figure 1E, PEGylated nBSA did not induce an obvious change in the concentration of O_2_. Native CAT was able to rapidly produce O_2_, while the PEGylated nCAT yielded a steady increase in the O_2_ concentration over time, both at pH 7.4 and 6.5. The generation of O_2_ bubbles in the H_2_O_2_ solutions (200 µM) was then recorded. It is clear from Figure 1F that both the native CAT and PEGylated nCAT could catalyze the decomposition of H_2_O_2_ to generate O_2_ bubbles, which is of great importance for the FUS application. Next, the enzyme activity after longer-term storage was investigated. From Figure S4 it can be observed that the PEGylated nCAT still kept 91% of its original activity after storage for 4 weeks, whereas the native CAT lost most of its activity under the same conditions. 21, 24. Enzyme digestion by protease activity also plays an important role during *in vivo* usage, and we therefore evaluated the protection of PEGylated nCAT against proteolysis. As shown in Figure S5, the PEGylated nCAT showed 90% of its initial activity after incubation with trypsin for 90 min, indicating the significant resistance to proteolysis by trypsin. Indeed, native CAT lost almost all its activity after the same period of incubation 25. Finally, since FUS is able to generate high operational temperatures (>50 ^o^C) which can disrupt the spatial conformation of the tetrameric CAT, we next examined the enzyme activity at this elevated temperature (50 ^o^C). As can be seen from Figure 1G, the PEGylated nCAT still retained 40 % of its original activity after incubation at 50 ^o^C for 30 min, while the native CAT lost almost all of its activity. The outstanding long-term, proteolytic and thermal stability of the PEGylated nCAT is due to the confinement of CAT within the surface shell, which prevents the enzyme from dissociating into subunits and which protects it from protease activity 21, 26.

### *In vitro* cytotoxicity

**Figure 2:**
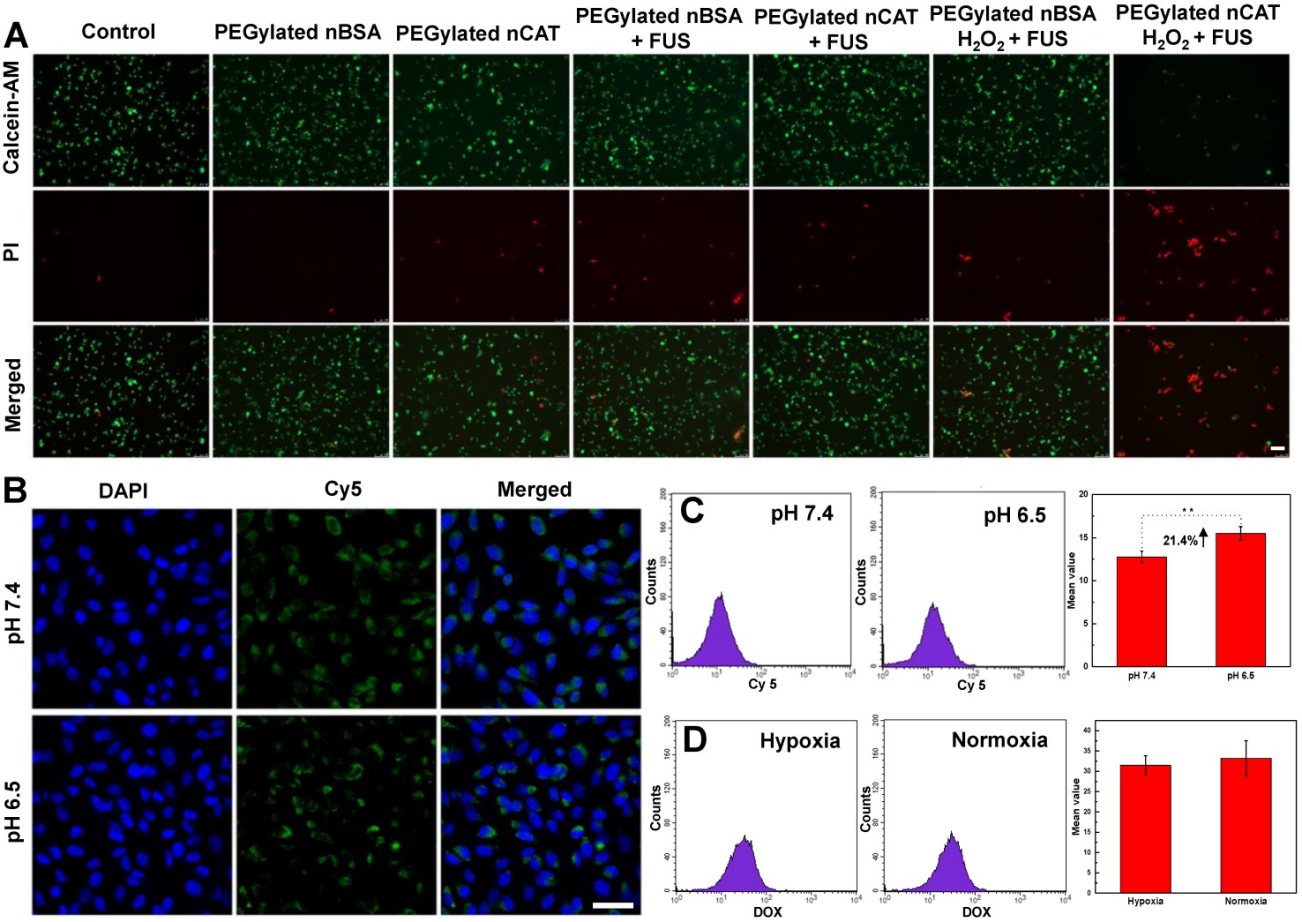
** (A)** Fluorescent images of 4T1 cells incubated under different treatment conditions. Calcein-AM and PI were employed to stain the living and dead cells, respectively. Scale bar: 100 µm. **(B)** CLSM images of 4T1 cells incubated with fluorescent PEGylated nCAT at pH 7.4 and pH 6.5 for 4 h. The nuclei were stained with Hoechst 33342. Scale bar: 50 µm. **(C)** FACS results of the uptake of PEGylated nCAT in 4T1 cells incubated at pH 7.4 and pH 6.5. PEGylated nCAT was labeled with Cy5. (D) FACS results of DOX uptake of 4T1 cells incubated with DOX (2 µg/mL) in the absence (hypoxia) or presence (normoxia) of PEGylated nCAT.

The toxicity of PEGylated nCAT toward mice embryonic fibroblast cells (NIH 3T3) and murine breast carcinoma cells (4T1) was assessed using a standard cell counting kit-8 (CCK-8) assay. We observed from Figure 1H that the cell viabilities of both NIH 3T3 and 4T1 cells incubated with PEGylated nCAT after 24 h were not affected at the PEGylated nCAT concentrations of 10, 25, 50, 75 and 100 µg/mL. This indicates that the PEGylated nCAT possesses favorable cytocompatibility which should not lead to damage to healthy tissues. We next explored the cytotoxicity of DOX under hypoxic and PEGylated nCAT-induced normoxic conditions. As can be seen from Figure 1I, DOX under hypoxic conditions displayed lower cytotoxicity to 4T1 cells, compared to normoxic conditions, which could be attributed to the hypoxia-induced drug resistance 16. FUS-induced cell apoptosis was further investigated using an ultrasonicator with a relatively low power of 0.3 W cm^-2^ and a treatment time of 10 sec. 4T1 cells after FUS irradiation were stained with Calcein-AM/propidium iodide (PI) (Calcein-AM for living cell staining and PI for dead cell staining) to observe the apoptosis using fluorescence microscopy (Figure 2A). It is clear that 4T1 cells treated with PEGylated nBSA or PEGylated nCAT in the presence or absence of FUS irradiation showed no obvious apoptosis. However, in presence of H_2_O_2_ (200 µM) and PEGylated nCAT, significant apoptosis was observed after FUS treatment. *In vitro* cytotoxicity assays thus demonstrate that the developed PEGylated nCAT showed no obvious toxicity to both healthy and cancer cells, but meanwhile could enhance the *in vitro* toxicity of DOX under hypoxic culture conditions, owing to the decomposition of H_2_O_2_ which thereby relieved the hypoxia. Moreover, the PEGylated nCAT were able to induce significant apoptosis under FUS treatment in presence of H_2_O_2_ owing to the generation of O_2_.

### *In vitro* cellular uptake

Confocal laser scanning microscopy (CLSM) and flow cytometry (FACS) were employed to explore the *in vitro* cellular uptake behavior of PEGylated nCAT at physiological pH of 7.4 and tumor microenvironment pH of 6.5. We can see from Figure 2B that the fluorescence intensity of 4T1 cells at pH 6.5 was slightly stronger than that at pH 7.4, indicating that 4T1 cells could take up higher amounts of PEGylated nCAT under slightly acidic pH than under physiological pH. The enhanced cellular uptake was further quantified with FACS to be 21.4% (Figure 2C). It is known that PEGylation on the surface of nanoparticles can prolong the circulation time, however, it also leads to a decreased cellular uptake 27. In this work, PEG is conjugated to the nCAT shell via an imine bond which is sensitive to slightly acidic pH 22, 28. The results can therefore be explained by de-PEGylation of nCAT (Figure 1A), which therefore improves the cellular uptake.

To check whether the treatment of PEGylated nCAT influences the uptake of DOX, we next used FACS to investigate the cellular uptake of DOX in presence or absence of PEGylated nCAT. As shown in Figure 2D, 4T1 cells displayed a similar amount of cellular uptake of DOX with or without treatment with PEGylated nCAT, suggesting that the PEGylated nCAT does not alter the uptake of DOX. This is important as this implies that a possible positive effect of nCAT on chemotherapy by relieving hypoxia will not be counteracted by a worse DOX uptake in the tumor tissue.

### *In vivo* photoacoustic imaging

**Figure 3:**
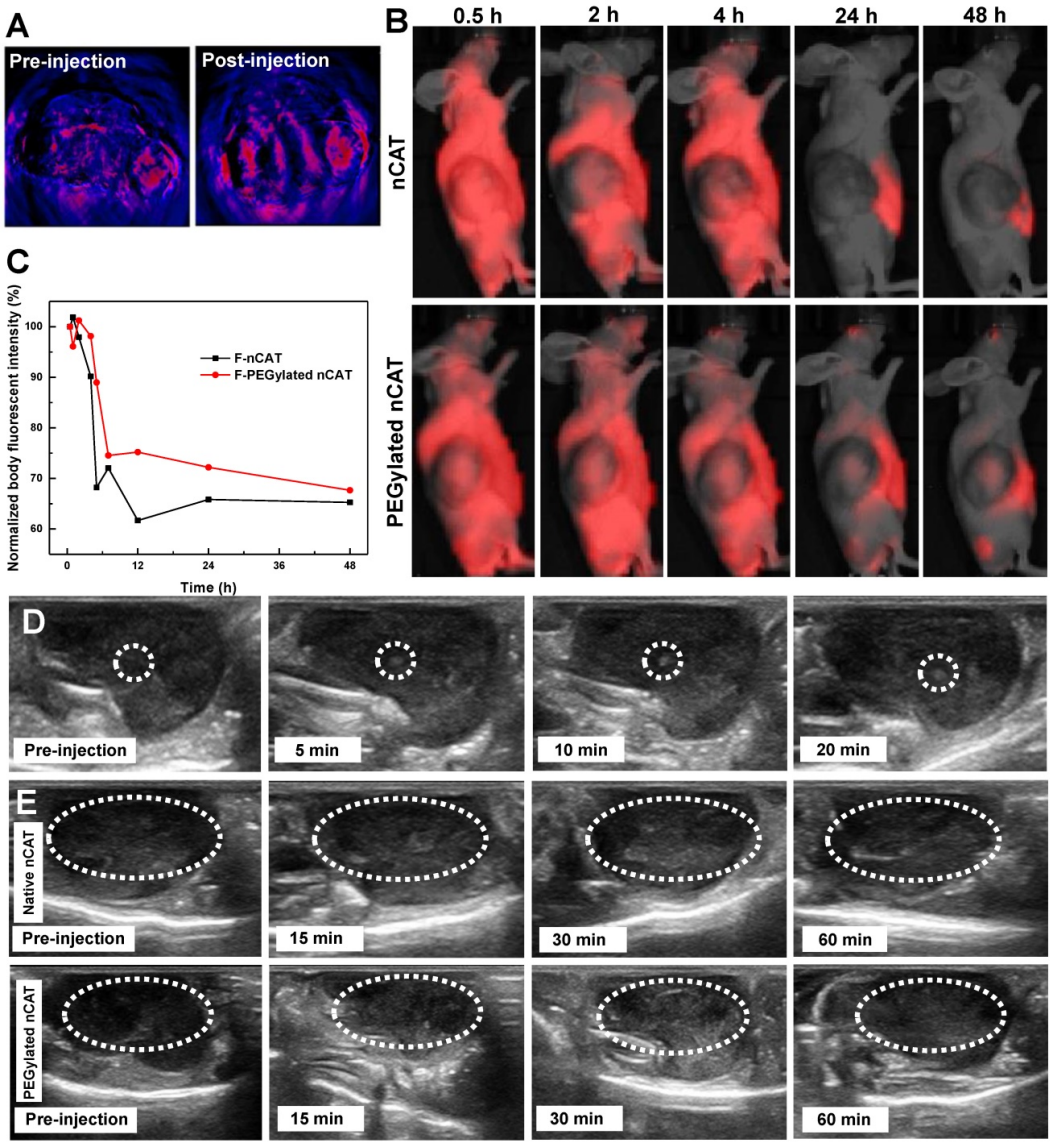
** (A)** Pseudo-colored *in vivo* photoacoustic imaging of 4T1 tumors before and after intratumoral injection of the PEGylated nCAT (20 µL, 1 mg/mL). **(B)** IVIS images of 4T1 tumor bearing nude mice at different time intervals after intravenous injection of fluorescent nCAT and PEGylated nCAT (200 µL, 1 mg/mL). **(C)** The normalized body fluorescent intensity of 4T1 tumor bearing nude mice at different time intervals after intravenous injection of nCAT and PEGylated nCAT (200 µL, 1 mg/mL). **(D)** *In vivo* US imaging of 4T1 tumors before and after intratumoral injection of the PEGylated nCAT (20 µL, 1 mg/mL) at different time intervals. **(E)** *In vivo* US imaging of 4T1 tumors before and after intravenous injection of native CAT and the PEGylated nCAT (200 µL, 1 mg/mL) at different time intervals.

To analyze the O_2_ levels of 4T1 tumors before and after intratumoral injection of the developed PEGylated nCAT, photoacoustic imaging was employed taking advantage of the difference in absorbance of oxygenated and deoxygenated hemoglobin at 850 and 750 nm, respectively. When the endogenous H_2_O_2_ was decomposed to yield an increased O_2_ concentration, the deoxygenated hemoglobin was re-oxygenated, thus resulting in an increased photoacoustic imaging signal. It is clear from Figure 3A that the O_2_ levels in the tumor region were increased after the treatment with PEGylated nCAT, indicating that the hypoxic tumor microenvironment was relieved via the CAT-mediated decomposition of the endogenous H_2_O_2_. This result indicates that also in *in vivo* situations, PEGylated nCAT can alleviate the hypoxic tumor microenvironment via the generation of O_2_ which should improve the therapeutic efficiency of DOX.

### *In vivo* circulation

Nude mice bearing xenografted 4T1 tumors were then employed to investigate the systematic circulation of nCAT and PEGylated nCAT. *In vivo* fluorescence imaging (IVIS) assays demonstrated that both the Cy5-labeled nCAT and PEGylated nCAT were able to be delivered into the 4T1 tumor and accumulated within the tumor region possibly via the passive enhanced permeability and retention (EPR) effect (Figure 3B and S6) 29, 30. It is worth to note that the PEGylated nCAT displayed an unexpected *in vivo* circulation time up to 48 h (Figure S7). In addition, the normalized body fluorescent intensity showed that the developed PEGylated nCAT displayed a longer blood half-life time (5.8 h) when compared to that of nCAT (4 h) (Figure 3C). This considerably prolonged circulation time holds much potential to optimally benefit from the particles' features regarding hypoxia relief, owing to the higher accumulation and extended retention time, which contribute to a higher generation of O_2_ from H_2_O_2_ produced in the tumor tissue.

### *In vivo* US imaging

In order to establish the positive effect on US imaging of PEGylated nCAT-mediated O_2_ production, US imaging of nude mice bearing 4T1 tumors after intratumoral injection of PEGylated nCAT (20 µL, 1 mg/mL) was conducted. It is clear from Figure S8 that no obvious contrast change was observed at the normal tissue injection site. The contrast enhancement at the tumor injection site was however detected right after the intratumoral injection, indicating the generation of O_2_ owing to the fast reaction between CAT and endogenous H_2_O_2_ (Figure 3D and S9). To further verify US imaging efficiency, US imaging and the corresponding mean gray values were then recorded at various time intervals after intravenous injection of native CAT and PEGylated nCAT (200 µL, 1 mg/mL) via the tail vein (Figure 3E and S10). As can be seen in Figure 3E, native CAT revealed a negligible US signal change in the tumor site after 60 min. The tumor region displayed a relatively low US signal before the injection and started to be brightened at 15 min post-injection of the PEGylated nCAT. The tumor tissue was brightest at 30 min post-injection which could be attributed to the highest accumulation of O_2_ bubbles within the tumor site. The US imaging then started to decline after 30 min which was possibly due to the consumption of endogenous H_2_O_2_. Therefore, we think that the endogenous H_2_O_2_-responsive O_2_ gas releasing strategy can be used as a contrast agent modality for US imaging of tumor models. Moreover, based on the *in vivo* US imaging, the FUS treatment was set at 30 min post-injection due to the highest accumulation of O_2_ bubbles.

### Combinational FUS ablation and chemotherapy

**Figure 4:**
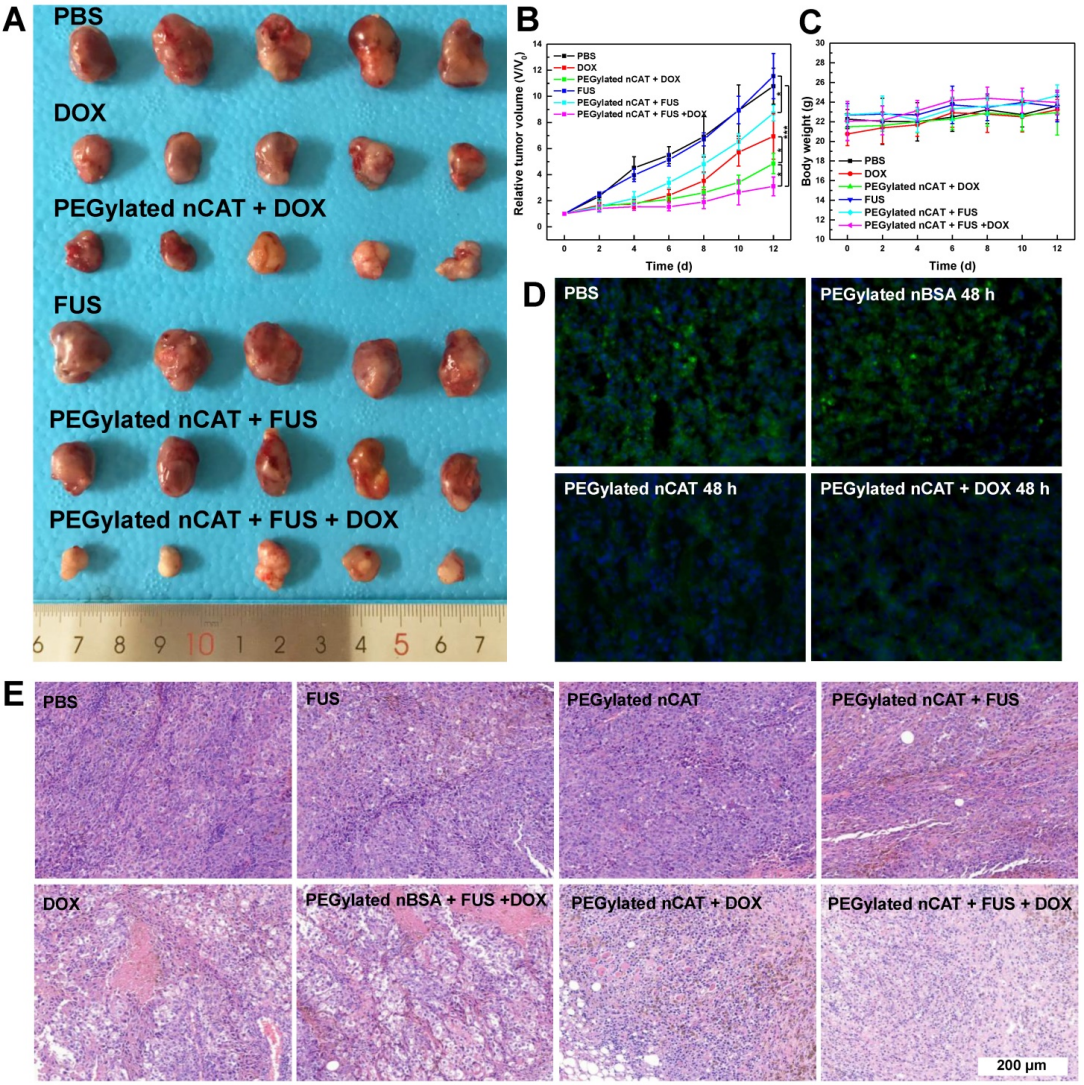
** (A)** Photograph of tumors after the *in vivo* therapy. The dosage of DOX and PEGylated nCAT is 5 mg/kg and 10 mg/kg, respectively. FUS was conducted with the settings of 1.5 W/cm^2^ and 50% duty circle for 3 min. **(B)** Tumor growth curves after the different treatments. Tumor volumes (V) were normalized to their initial values (V_0_). **(C)** Mice body weight changes of different groups over 12 d. **(D)** Immunofluorescence staining images of tumor slices after different treatments for 48 h. The cell nuclei and hypoxia areas were stained by DAPI (blue) and antipimonidazole antibody (green), respectively. **(E)** H&E staining of tumor slices taken at day 12 after different treatments.

After demonstrating the effective H_2_O_2_-responsive O_2_ release mediated by PEGylated nCAT, *in vivo* FUS ablation combined with DOX chemotherapy was investigated using a 4T1 tumor bearing nude mouse model to verify the capability of PEGylated nCAT to serve as a synergistic agent. 30 nude mice bearing 4T1 tumors with size of ≈ 100 mm^3^ were randomly divided into 6 groups which were treated respectively witj: (1) PBS, (2) PEGylated nCAT, (3) Free DOX, (4) PEGylated nCAT + DOX, (5) FUS, (6) PEGylated nCAT + FUS, (7) PEGylated nBSA + FUS +DOX and (8) PEGylated nCAT + FUS +DOX, with the corresponding DOX and PEGylated nCAT dose of 5 mg/kg and 10 mg/kg, respectively. The therapeutic effectiveness was evaluated by monitoring the tumor size changes, and the representative tumor images of each group were recorded after the therapy (Figure 4A, 4B, S11A and S11B). It was found that the mice in the PBS, PEGylated nCAT and FUS groups displayed a similar tumor growth rate. It is interesting that the FUS treatment alone under the experimental conditions did not lead to potential tumor ablation due to the poor-acoustic tumor environment. This is further demonstrated by the tumor position temperature increase of ≈5^o^C, which could not generate obvious harmful effects on the tumor cells (Figure S12). The treatment with FUS after injection of PEGylated nCAT offered a better tumor inhibition effect, compared to the treatment with FUS alone, which can be attributed to the generated O_2_ via the PEGylated nCAT-mediated H_2_O_2_ decomposition, which improved the acoustic tumor environment, thus producing more heat to kill the cancer cells (Figure S12). Tumor growth in the group with only DOX administration and PEGylated nBSA + FUS + DOX displayed a similar partial inhibition trend. Notably, the treatment of PEGylated nCAT + DOX further suppressed the tumor growth, which was attributed to the enhanced toxicity of DOX owing to the relieved hypoxia (Figure 4D). Most importantly, the combined treatment of FUS +DOX after the injection of PEGylated nCAT provided the most effective inhibition effect on tumor growth over all other groups, which could be attributed to the relieved hypoxia and the high tumor site temperature. The hematoxylin-eosin (H&E) staining of the tumor slices also revealed that combined treatment of FUS +DOX after the injection of PEGylated nCAT showed the highest level of cancer cell apoptosis compared to the other groups (Figure 4E), futher demonstrating the observed therapeutic effects based on tumor volume. Based on the *in vivo* therapy results, we believe that the developed PEGylated nCAT are able to significantly improve the therapeutic efficacy of the combined FUS ablation and DOX chemotherapy.

### Biocompatibility evaluation

Mice body weight changes were recorded in all groups to estimate the *in vivo* toxicity of the developed PEGylated nCAT. It is clear from Figure 4C and S12 that no obvious changes of mice body weight were observed in all groups following the process of *in vivo* therapy. H&E staining was further conducted for histological examinations. As shown in Figure S13, no significant pathological abnormalities such as inflammatory infiltrate, morphological changes and necrosis were found in the slices of heart, liver, spleen, lung, and kidney after intravenous injection of the PEGylated nCAT after 30 d, when compared to the control group without any treatment. This demonstrates that the developed PEGylated nCAT display satisfactory biocompatibility to the mouse organs.

In this work, we have developed a novel catalytic PEGylated nCAT as a 1) contrast agent for US imaging 2) strengthening agent for FUS ablation and 3) normoxia inducer to enhance chemotherapeutic efficacy. The nanoparticle was constructed by encapsulation of CAT within a polymer shell followed by surface decoration with a pH-sensitive PEG layer. Such PEGylated nCAT induced endogenous H_2_O_2_ decomposition to continuously release O_2_ bubbles for sustained contrast enhancement of US imaging and augmented tumor ablation under FUS exposure. Furthermore, the pH-responsive surface PEG significantly prolonged the blood circulation time whereas it bypassed the PEGylation-induced decreased cellular uptake. More importantly, this developed PEGylated nCAT alleviated the hypoxic tumor microenvironment, thus solving hypoxia-induced drug resistance to improve the therapeutic outcome of DOX treatment. This theranostic strategy which allows to simultaneously address several limitations of US-guided FUS ablation and conventional chemotherapy holds much promise in the application of cancer therapy.

## Materials and Methods

### Materials

All materials were purchased from Sigma-Aldrich unless otherwise noted. Dulbecco's modified eagle's medium (DMEM), RPMI 1640 medium, trypsin, penicillin/streptomycin (P/S), fetal bovine serum (FBS), phosphate buffered saline (PBS), Hoechst 33342, and Pierce™ BCA protein assay kit were purchased from ThermoFisher Scientific. Cell counting kit-8 (CCK-8) was acquired from 7sea Biotech. Co., Ltd. (Shanghai, China). Cyanine5 NHS ester (Cy5-NHS) was purchased from Lumiprobe GmbH (Germany). Calcein-AM and propidium iodide (PI) were obtained from Dojindo Laboratories (Shanghai, China). All chemicals and materials were used without further purification.

### Instruments

Malvern Z90 Zetasizer equipped with a 633 nm laser and an avalanche photodiode detector was used to characterize the hydrodynamic size. ^1^H NMR spectra were recorded using a Bruker 400 NMR spectrometer. Transmission Electron Microscopy (TEM) images were recorded on a FEI Tecnai 20 (type Sphera). Scanning electron microscopy (SEM) images were characterized by FEI Quanta 200 3D FEG. Ultraviolet-visible Spectroscopy (UV/Vis) absorbance was recorded on a Jasco V-650 UV/Vis spectrometer. The O_2_ concentration was determined using a portable meter MultiLine® Multi 3510 IDS. Microplate reader (Safire^2^, TECAN) was employed for the CCK-8 assay. Fluorescence images were observed and captured by QuantaMaster-40 fluorescence S-3 spectrophotometer and Leica TCS SP5X. A Becton Dickinson FACScan flow cytometer was used to determine the cellular uptake. The photoacoustic images were captured by an *in vivo* photoacoustic system (MSOT inVision, iThera Medical). The *in vivo* circulation behavior was recorded by an *in vivo* imaging system (PerkinElmer IVIS Lumina Series III). The ultrasound imaging was performed on a Mindray resona 7. FUS treatment was conducted using Intelect® Mobile Ultrasound. Tumor position temperature changes were collected by a thermal imager (FLIR A300, IRS Systems Inc.) coupled with an infrared camera.

### Synthesis of nCAT

CAT (100 mg) was dispersed into 45 mL of PBS (50 mM, pH 7.4) and 37.9 mg of the N hydroxy succinimide ester of acrylic acid (NAS, molar ratio of NAS to the amine group of CAT, 5:1) dissolved in 5 mL of DMSO was added to the above solution. The reaction was kept at room temperature (RT) for 4 h followed by dialysis (MWCO = 12 -14 kDa) against PBS at 4 ^o^C to remove the unreacted NAS and DMSO. The cloudy acryloylated CAT solution was then filtered through an Acrodisc® syringe filter (0.22 µm) to obtain a clear solution. The protein concentration was determined using a Pierce™ BCA protein assay kit and the solution was stored at 4 ^o^C for further use. The average number of acryloyl groups conjugated onto each enzyme was determined with a fluorescamine assay according to the literature reported 20. Acryloylated bovine serum albumin (BSA) was synthesized following the same procedure.

50 mL of acryloylated CAT solution (1 mg/mL) was then degassed with nitrogen for 30 min before use. Acrylamide (AA, 42.6 mg in 1 mL degassed MQ water), N-(3-aminopropyl)methacrylamide hydrochloride (APM, 35.7 mg in 1 mL DMSO) and N, N'-methylenebisacrylamide (MBA, 12.3 mg in 1 mL DMSO) were then added. The radical polymerization on the surface of the acryloylated CAT was initiated by adding ammonium persulfate (APS, 45.6 mg in 1 mL degassed MQ water) and N, N, N', N'-tetramethylethylenediamine (TEMED, 60 µL in 1 mL MQ water). The reaction was allowed to proceed for 4 h in a nitrogen atmosphere and cooled with an ice bath. Finally, dialysis (MWCO = 12 -14 kDa) against PBS at 4 ^o^C was conducted to remove unreacted monomers and initiators. The synthesized nCAT was stored at 4 ^o^C for further use. BSA encapsulated nanoparticles (nBSA) were synthesized as a control using the same method.

Cy5 labeled CAT was prepared for the fluorescence assays. Briefly, 15 mg of CAT was first dissolved into 7 mL of PBS buffer (50 mM, pH 7.4) and 0.4 mg of Cy5-NHS ester dissolved in 0.5 mL of DMSO was then added. The reaction was carried out at 37°C for 4 h. Labeled CAT was then dialyzed against PBS (MWCO = 12 -14 kDa). The fluorescent nCAT was synthesized following the same procedure mentioned above.

### Synthesis of PEGylated nCAT

Aldehyde group terminated PEG was first synthesized according to the reported method with slight modifications 31. Firstly, PEG_5k_ (10 g, 2 mmol), 4-carboxybenzaldehyde (1.5 g, 10 mmol), N-(3-dimethylaminopropyl)-N'-ethylcarbodiimide hydrochloride (3.85 g, 20 mmol) and 4-(dimethylamino)pyridine (0.06 g, 0.5 mmol) were dissolved in 100 mL dichloromethane and stirred for 48 h at 25°C. After the reaction was completed, the solution was concentrated by a rotary evaporator, and then the mixture was washed for 5 times with saturated NaCl solution and another 3 times with 5% NaCl solution, respectively. Subsequently, the organic layer was collected and anhydrous magnesium sulfate as dehydration agent was added. After filtration, the filtrate was concentrated and precipitated twice with excess diethyl ether. The final product was dried under vacuum at room temperature overnight. The product was characterized by ^1^H NMR spectroscopy in CDCl_3_.

The synthesized nCAT was then mixed with the aldehyde-terminated PEG (molar ratio of CAT to PEG, 1:100) in PBS and stirred at RT from 4 h. After dialysis against PBS at 4 ^o^C, the final product was obtained and stored at 4 ^o^C for further use.

To verify the cleavage of PEG from the PEGylated nCAT, the PEGylated nCAT solution was dialysed against MES buffer (pH 6.5) for 1 d (MWCO = 12 -14 kDa). The dialysis solution containing the cleaved PEG was collected and concentrated with Amicon tube (MWCO = 3 kDa). After removing the MES salts via buffer exchange, the solution was freeze-dried. The freeze-dried sample was characterized by ^1^H NMR spectroscopy in CDCl_3_.

### Evaluation of enzyme activity

The catalytic activities of the acryloylated CAT and PEGylated nCAT were measured by monitoring the decomposition of H_2_O_2_ at the UV wavelength of 240 nm. Briefly, the H_2_O_2_ solution was diluted with PBS (50 mM, pH 7.4) to ensure an A_240_ between 0.550 and 0.520. Fresh CAT, acryloylated CAT or PEGylated nCAT solutions were prepared at a CAT concentration of 50 µg/mL. 2.9 mL of diluted H_2_O_2_ solution was pipetted into a quartz cuvette and placed into the spectrophotometer. 0.1 mL of CAT-containing solution was added into the quartz cuvette and immediately mixed by inversion. The absorbance of H_2_O_2_ at 240 nm was monitored for ≈ 180 seconds. All of the assays were performed at least in triplicate. The relative enzyme activity of acryloylated CAT and PEGylated nCAT compared to fresh CAT was calculated by measuring the time required for the A_240_ to decrease from 0.45 to 0.40. The oxygen concentration change in H_2_O_2_ solution (500 µM) incubated with CAT, PEGylated nBSA or PEGylated nCAT was monitored using a portable oxygen meter.

To investigate the catalytic activity of enzyme in presence of protease, native CAT and PEGylated nCAT were incubated with trypsin (44 µM) at 40 ^o^C for 90 min, the enzyme activity was then determined as mentioned above. For the thermal stability assay, native CAT and nCAT were incubated at 50 °C for different times, and the enzyme activity was measured using the aforementioned method.

### Cell culture

Mice embryonic fibroblast cells (NIH 3T3) were cultured in DMEM supplemented with 10% FBS and 1% P/S. Murine breast carcinoma cells (4T1) were cultured in RPMI-1640 medium supplemented with 10% FBS and 1% P/S.

### Evaluation of cytotoxicity

Biocompatibility of PEGylated nCAT towards healthy (NIH 3T3) and cancerous (4T1) cell lines was assessed using standard CCK-8 assays referring to literature reported 32. Briefly, cells were first seeded in a 96-well plate at a density of 5 000 cells/well and cultured overnight. Cells were then incubated with fresh medium containing different concentrations of PEGylated nCAT (0, 10, 25, 50, 75 and 100 µg/mL) and cultured for another 24 h. After rinsing 3 times with PBS, the cells were incubated with 100 μL of culture medium without FBS but supplemented with 10% CCK-8 for 2 h. The absorbance at 450 nm was recorded on the microplate reader. For each sample concentration, 5 parallel experiments were analyzed to give a mean value and standard deviation.

FUS-induced cell death was explored using a fluorescence assay. 4T1 cells were seeded in a 6-well plate at a density of 50 000 cells/well and cultured overnight. Culture medium containing PEGylated nBSA or PEGylated nCAT (protein concentration 50 µg/mL) was then added into the plate to replace the old medium. After incubation for 4 h, the cells were further cultured in fresh medium in presence or absence of H_2_O_2_ (200 µM). Afterward, ultrasound couplant was pasted to the bottom of the well plate and FUS treatment was conducted with the settings of 0.3 W/cm^2^ and 50% duty circle for 10 sec. After removing the couplant, Calcein-AM and propidium iodide (PI) were employed to stain the dead and living cells, respectively. The fluorescent images were observed and captured using fluorescence microscopy.

To investigate hypoxia-induced drug resistance, the toxicity of DOX was evaluated at hypoxic and normoxic conditions using the CCK-8 assay as aforementioned. 4T1 cells were first seeded in a 96-well plate at a density of 5 000 cells/well and cultured overnight in a hypoxic cell incubator (5% CO_2_, 94% N_2_, 1%O_2_). Fresh medium containing H_2_O_2_ (200 µM) and different DOX concentrations (0, 0.25, 0.5, 1, 2.5, 7.5 and 10 µg/mL) were then added. To construct a normoxic culture condition, PEGylated nCAT (50 µg/mL) was added to the culture medium. After culturing for another 24 h, cell viabilities were recorded using the CCK-8 assay.

### *In vitro* cellular uptake

The cellular uptake of PEGylated nCAT in 4T1 cells at physiological pH of 7.4 and tumor microenvironment pH of 6.5 were measured by CLSM and FACS. Briefly, PEGylated nCAT (50 µg/mL) were first incubated with 4T1 for 4 h. The culture medium was then removed by washing the cells with PBS buffer 3 times. The confocal images and fluorescent signal were then detected. To explore the influence of PEGylated nCAT towards the uptake of DOX, 4T1 cells were cultured with culture medium containing DOX (2 µg/mL) in presence or absence of PEGylated nCAT (50 µg/mL) for 2 h and the fluorescent signal was determined by FACS.

### *In vivo* circulation of PEGylated nCAT

Animal experiments were performed according to protocols approved by the ethical committee of Shanghai General Hospital, and all handlings of mice were also in accordance with the regulations of the National Ministry of Health. Male 4- to 6-week-old BALB/c nude mice (20-25 g, Shanghai Slac Laboratory Animal Center, Shanghai, China) were used to establish a xenografted tumor bearing model using a method reported in the literature 33. The *in vivo* circulation behavior of PEGylated nCAT was explored using 4T1 tumor bearing nude mice. Briefly, 200 µL of PEGylated nCAT solution was intravenously injected into the 4T1 tumor bearing mice via the tail vein. The *in vivo* fluorescent images were captured at different time intervals using an *in vivo* imaging system.

### *In vivo* photoacoustic imaging

4T1 tumor bearing nude mice were intratumorally injected with PEGylated nCAT (20 µL, 1 mg/mL) and imaged by an *in vivo* photoacoustic imaging system before and after the injection to evaluate tumour O_2_ levels.

### US imaging of 4T1 tumor model

4T1 tumor bearing nude mice were used to investigate the US imaging of the tumor via intratumoral and intravenous injection. For the intratumoral injection, 20 µL of PEGylated nCAT solution (1 mg/mL) was injected into the tumor and the injection site was monitored by B-mode US imaging using Mindray resona 7. For the intravenous injection, 200 µL of PEGylated nCAT solution (1 mg/mL) was injected into the mice via the tail vein and the tumor site was monitored by B-mode US imaging using Mindray resona 7. The gray scale values were analyzed using software ImageJ.

### Immunofluorescent staining

Immunofluorescence staining of tumor slices was conducted to explore the tumor hypoxia. Briefly, 4T1 tumor-bearing nude mice were intravenously injected with 200 µL of PBS, PEGylated nBSA or PEGylated nCAT (1 mg/mL). After 48 h, the tumors were surgically excised 90 min after intraperitoneal injection of pimonidazole hydrochloride (0.6 mg/mouse). Antipimonidazole mouse monoclonal primary antibodies and Alex 488-conjugated goat antimouse secondary antibodies were employed for staining and fluorescence imaging.

### Combinational FUS and chemotherapy

4T1 tumor bearing nude mice were randomly divided into 6 groups which were treated with: (1) PBS, (2) Free DOX, (3) PEGylated nCAT + DOX, (4) FUS, (5) PEGylated nCAT + FUS and (6) PEGylated nCAT + FUS +DOX with the corresponding DOX and PEGylated nCAT dose of 5 mg/kg and 10 mg/kg, respectively. Briefly, PEGylated nCAT solution (1 mg/mL) was intravenously injected into 4T1 tumor bearing nude mice via the tail vein. After 30 min, ultrasound couplant was pasted to the tumor site and FUS treatment was conducted with the settings of 1.5 W/cm^2^ and 50% duty circle for 3 min. The tumor position temperature change was recorded before and after FUS treatment by a thermal imager.DOX in saline was thereafter intravenously injected into the mice. The therapy process was repeated one more time after 3 d. The 4T1 tumor volume and body weight were monitored every 2 d. After 12 d, the tumors collected from different groups were stained with H&E.

### *In vivo* biocompatibility evaluation

PEGylated nCAT solution (200 μL, I mg/mL) was intravenously administered to the healthy mice. After 30 d, the mice were sacrificed and the major organs including heart, liver, spleen, lung, and kidney were harvested for H&E staining. In addition, the mice without any treatment were used as the control.

## Supplementary Material

Supplementary figures and tables.

## References

[1] Yildirim A, Blum NT, Goodwin AP (2019). Colloids, nanoparticles, and materials for imaging, delivery, ablation, and theranostics by focused ultrasound (FUS). Theranostics.

[2] Curley CT, Sheybani ND, Bullock TN, Price RJ (2017). Focused ultrasound immunotherapy for central nervous system pathologies: Challenges and opportunities. Theranostics.

[3] Devarakonda SB, Myers MR, Lanier M, Dumoulin C, Banerjee RK (2017). Assessment of gold nanoparticle-mediated-enhanced hyperthermia using MR-guided high-intensity focused ultrasound ablation procedure. Nano Lett.

[4] Zhang N, Cai XJ, Gao W, Wang RH, Xu CY, Yao YZ (2016). A multifunctional theranostic nanoagent for dual-mode image-guided HIFU/chemo-synergistic cancer therapy. Theranostics.

[5] Zhou Y, Wang Z, Chen Y, Shen H, Luo ZY, Li A (2013). Microbubbles from gas-generating perfluorohexane nanoemulsions for targeted temperature-sensitive ultrasonography and synergistic hifu ablation of tumors. Adv Mater.

[6] Liu T, Zhang N, Wang Z, Wu M, Chen Y, Ma M (2017). Endogenous catalytic generation of O_2_ bubbles for *in situ* ultrasound-guided high intensity focused ultrasound ablation. ACS Nano.

[7] Wang X, Niu D, Li P, Wu Q, Bo X, Liu B (2015). Dual-enzyme-loaded multifunctional hybrid nanogel system for pathological responsive ultrasound imaging and T_2_-weighted magnetic resonance imaging. ACS Nano.

[8] Yang F, Hu S, Zhang Y, Cai XJ, Huang Y, Wang F (2012). A hydrogen peroxide-responsive O2 nanogenerator for ultrasound and magnetic-resonance dual modality imaging. Adv Mater.

[9] Hu D, Chen Z, Sheng Z, Gao D, Yan F, Ma T (2018). A catalase-loaded hierarchical zeolite as an implantable nanocapsule for ultrasound-guided oxygen self-sufficient photodynamic therapy against pancreatic cancer. Nanoscale.

[10] Malone CrD, Fetzer DT, Lux J, Mattrey RF (2019). Catalase-Containing Silica Particles as Ultrasound-Based Hydrogen Peroxide Sensors to Determine Infected From Noninfected Fluid Collections in Humans.

[11] Olson ES, Orozco J, Wu Z, Malone CD, Yi B, Gao W (2013). Toward *in vivo* detection of hydrogen peroxide with ultrasound molecular imaging. Biomaterials.

[12] van der Meel R, Vehmeijer LJC, Kok RJ, Storm G, van Gaal EVB (2013). Ligand-targeted particulate nanomedicines undergoing clinical evaluation: Current status. Adv Drug Deliv Rev.

[13] Deavall DG, Martin EA, Horner JM, Roberts R (2012). Drug-induced oxidative stress and toxicity. J Toxicol.

[14] Shin DH, Choi Y-J, Park J-W (2014). SIRT1 and AMPK mediate hypoxia-induced resistance of non-small cell lung cancers to cisplatin and doxorubicin. Cancer Res.

[15] Frederiksen LJ, Siemens DR, Heaton JP, Maxwell LR, Adams MA, Graham CH (2003). Hypoxia induced resistance to doxorubicin in prostate cancer cells is inhibited by low concentrations of glyceryl trinitrate. J Urol.

[16] Huang CC, Chia WT, Chung MF, Lin KJ, Hsiao CW, Jin C (2016). An implantable depot that can generate oxygen *in situ* for overcoming hypoxia-induced resistance to anticancer drugs in chemotherapy. J Am Chem Soc.

[17] Maleki T, Cao N, Song S, Kao C, Ko S, Ziaie B (2011). An ultrasonically powered implantable micro-oxygen generator (IMOG). IEEE Trans Biomed Eng.

[18] Luo ZY, Tian H, Liu LL, Chen ZK, Liang RJ, Chen Z (2018). Tumor-targeted hybrid protein oxygen carrier to simultaneously enhance hypoxia-dampened chemotherapy and photodynamic therapy at a single dose. Theranostics.

[19] Zhang W, Li S, Liu X, Yang C, Hu N, Dou L (2018). Oxygen-generating MnO2 nanodots-anchored versatile nanoplatform for combined chemo-photodynamic therapy in hypoxic cancer. Adv Funct Mater.

[20] Liu Y, Du J, Yan M, Lau MY, Hu J, Han H (2013). Biomimetic enzyme nanocomplexes and their use as antidotes and preventive measures for alcohol intoxication. Nat Nanotechnol.

[21] Liu L, Yu W, Luo D, Xue Z, Qin X, Sun X (2015). Catalase nanocapsules protected by polymer shells for scavenging free radicals of tobacco smoke. Adv Funct Mater.

[22] Guan X, Guo Z, Lin L, Chen J, Tian H, Chen X (2016). Ultrasensitive ph triggered charge/size dual-rebound gene delivery system. Nano Lett.

[23] Zhang J, Chi Q, Zhang B, Dong S, Wang E (1998). Molecular characterization of beef liver catalase by scanning tunneling microscopy. Electroanalysis.

[24] Riccardi CM, Cole KS, Benson KR, Ward JR, Bassett KM, Zhang YR (2014). Toward "stable-on-the-table" enzymes: Improving key properties of catalase by covalent conjugation with poly(acrylic acid). Bioconjugate Chem.

[25] Zhang X, Chen W, Zhu X, Lu Y (2017). Encapsulating therapeutic proteins with polyzwitterions for lower macrophage nonspecific uptake and longer circulation time. ACS Appl Mater Interfaces.

[26] Du Y, Gao J, Zhou L, Ma L, He Y, Huang Z (2017). Enzyme nanocapsules armored by metal-organic frameworks: A novel approach for preparing nanobiocatalyst. Chem Eng J.

[27] Pelaz B, del Pino P, Maffre P, Hartmann R, Gallego M, Rivera-Fernández S (2015). Surface functionalization of nanoparticles with polyethylene glycol: Effects on protein adsorption and cellular uptake. ACS Nano.

[28] Guan X, Guo Z, Wang T, Lin L, Chen J, Tian H (2017). A pH-responsive detachable peg shielding strategy for gene delivery system in cancer therapy. Biomacromolecules.

[29] Ojha T, Pathak V, Shi Y, Hennink WE, Moonen CTW, Storm G (2017). Pharmacological and physical vessel modulation strategies to improve EPR-mediated drug targeting to tumors. Adv Drug Deliv Rev.

[30] Arranja AG, Pathak V, Lammers T, Shi Y (2017). Tumor-targeted nanomedicines for cancer theranostics. Pharmacol Res.

[31] Zhao C, Deng H, Xu J, Li S, Zhong L, Shao L (2016). “Sheddable” PEG-lipid to balance the contradiction of PEGylation between long circulation and poor uptake. Nanoscale.

[32] Sun W, Thies S, Zhang J, Peng C, Tang G, Shen M (2017). Gadolinium-loaded poly(n-vinylcaprolactam) nanogels: Synthesis, characterization, and application for enhanced tumor mr imaging. ACS Appl Mater Interfaces.

[33] Zhu J, Peng C, Sun W, Yu Z, Zhou B, Li D (2015). Formation of iron oxide nanoparticle-loaded γ-polyglutamic acid nanogels for MR imaging of tumors. J Mater Chem B.

