# Supplementary material for: Single enzyme loaded nanoparticles for combinational ultrasound-guided focused ultrasound ablation and hypoxia-relieved chemotherapy
Source: Theranostics. 2019 Oct 17;9(26):8048–60. doi: 10.7150/thno.37054 (PMC6857069; doi:10.7150/thno.37054)
Supplement: Supplementary file 1 — Supplementary figures and tables. [file thnov09p8048s1.pdf]

Supporting information

Single enzyme loaded nanoparticles for combinational ultrasound-guided focused ultrasound ablation and hypoxia-relieved chemotherapy

Jianzhi Zhu[1,2*], Zhicong Li[3*], Changchang Zhang[2], Lizhou Lin[4], Shoupeng Cao[1], Hailong Che[1], Xiangyang Shi[2✉], Han Wang[3✉] and Jan C. M. van Hest[1✉]

1 Bio-Organic Chemistry, Institute for Complex Molecular Systems, Eindhoven University of Technology, 5600 MB Eindhoven, the Netherlands.

2 State Key Laboratory for Modification of Chemical Fiber and Polymer Materials, College of Chemistry, Chemical Engineering and Biotechnology, Donghua University, Shanghai 201620, People's Republic of China.

3 Department of Radiology, Shanghai General Hospital, Shanghai Jiao Tong University School of Medicine, Shanghai 20080, People's Republic of China.

4 Department of Ultrasound, Shanghai General Hospital, Shanghai Jiaotong University School of Medicine, Shanghai 20080, People's Republic of China.

* These authors contributed equally to this work.

✉ Corresponding authors. E-mail: xshi@dhu.edu.cn (X. Shi), han.wang@shsmu.edu.cn (H. Wang) and j.c.m.v.hest@tue.nl (J. C. M. van Hest)

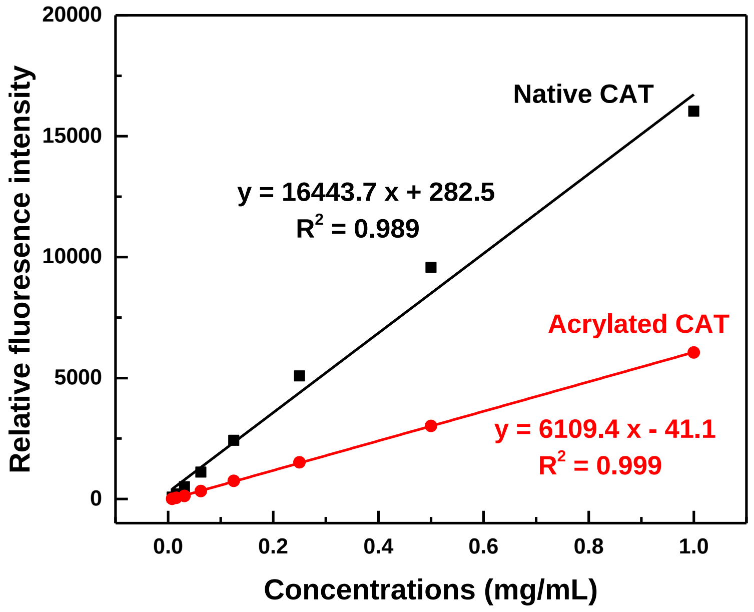

Figure S1. Fluorescamine assay of native CAT and acrylated CAT.

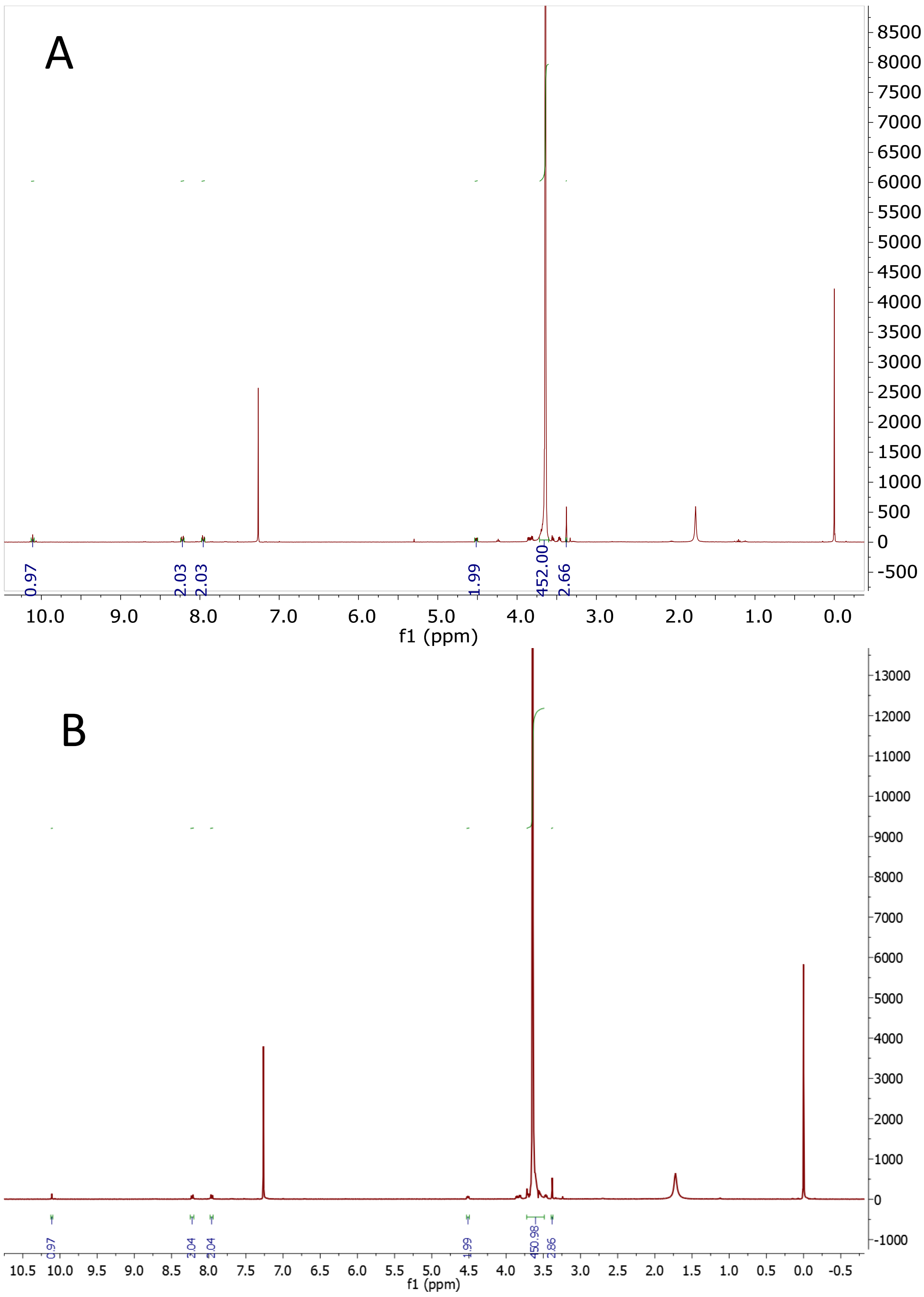

Figure S2. (A) ^{1}HNMR spectrum of the synthesized mPEG$_{5k}$-CHO. (B) ^{1}HNMR spectrum of the cleaved mPEG$_{5k}$-CHO.

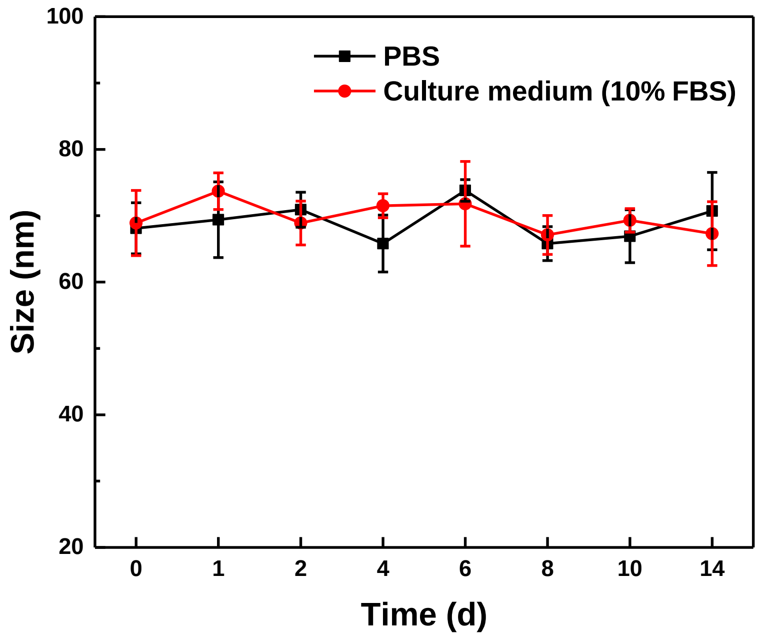

Figure S3. Hydrodynamic size change of the developed PEGylated nCAT in PBS and culture medium over a period of 14 d.

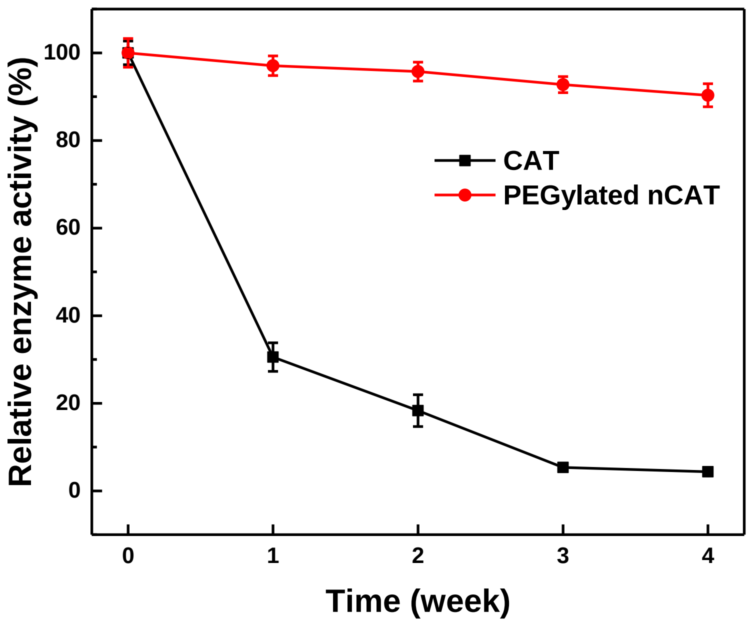

Figure S4. Relative enzyme activity of native CAT and PEGylated nCAT during a storage period of 4 weeks at 4°C.

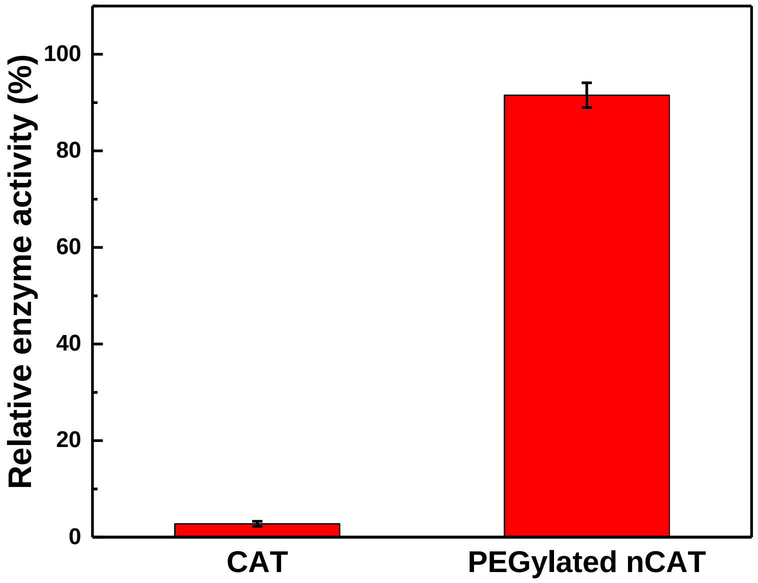

Figure S5. Relative enzyme activity of native and PEGylated nCAT after incubation with trypsin (44 μM).

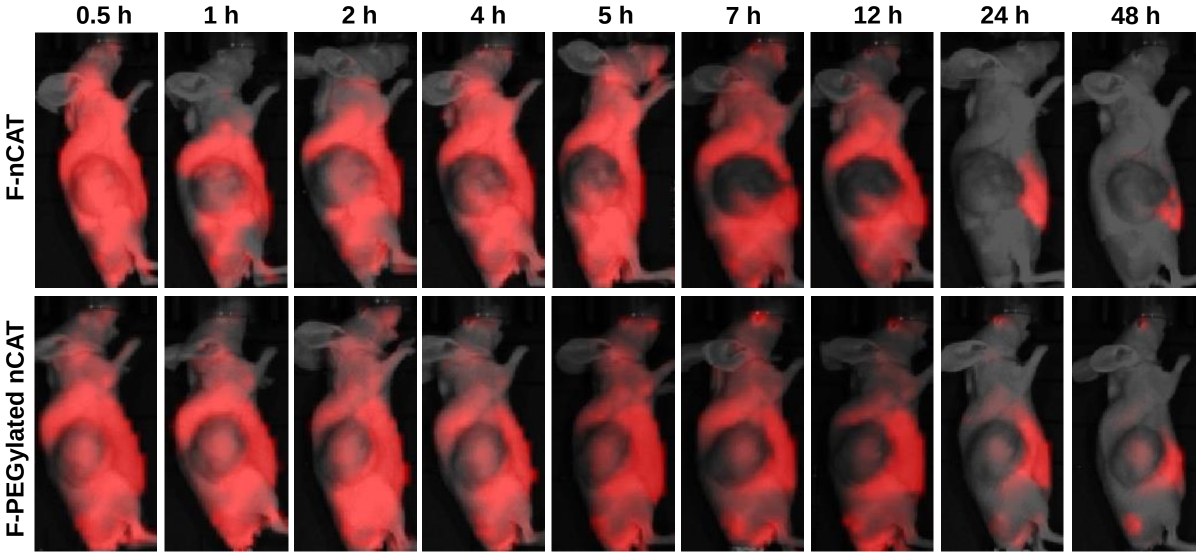

Figure S6. IVIS images of 4T1 tumor bearing nude mice at different time intervals after intravenous injection of nCAT and PEGylated nCAT (200 µL, 1 mg/mL).

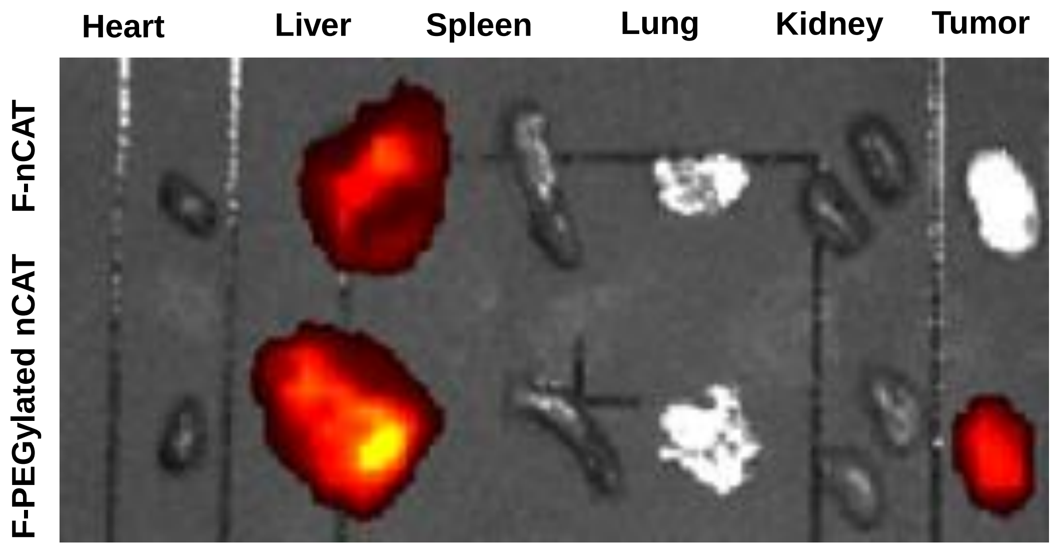

Figure S7. In vitro fluorescent images of organs after intravenous injection of nCAT and PEGylated nCAT (200 µL, 1 mg/mL) for 48 h.

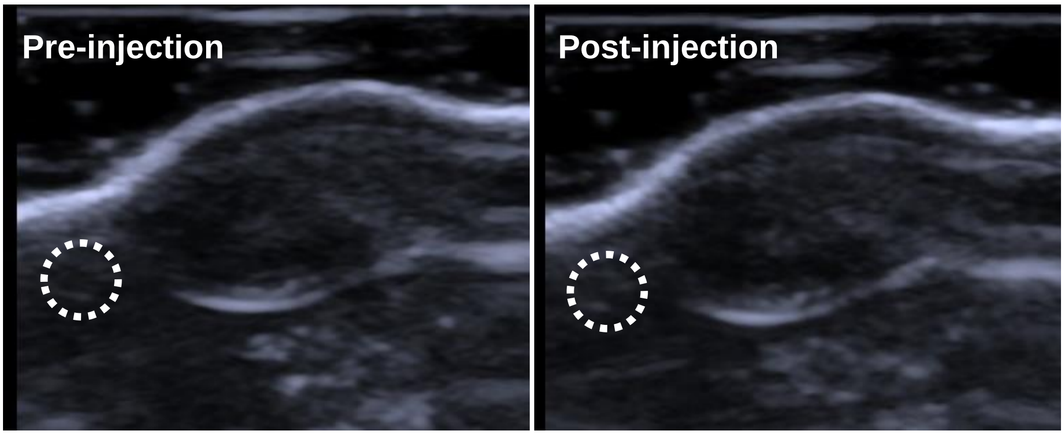

Figure S8. In vivo US imaging of normal tissues pre- and 10 min post-injection of the PEGylated nCAT (20 μL, 1 mg/mL).

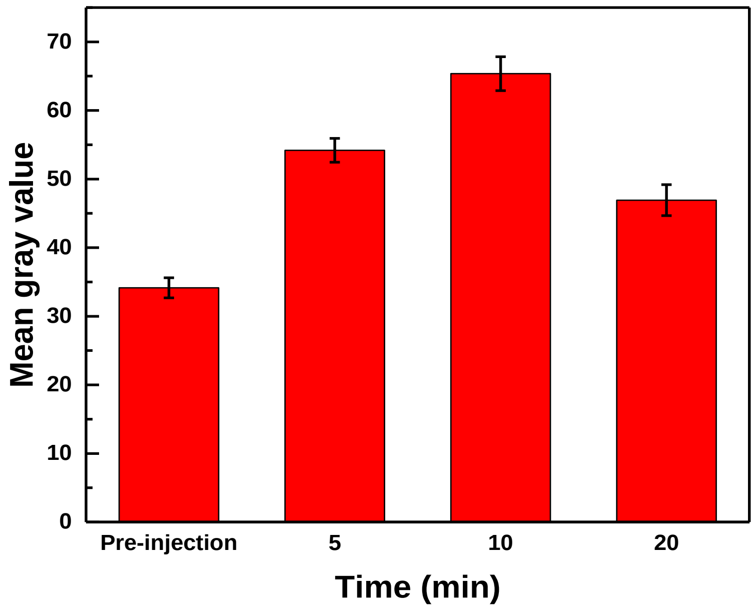

Figure S9. Mean gray values of the injection site before and after intratumoral injection of the PEGylated nCAT.

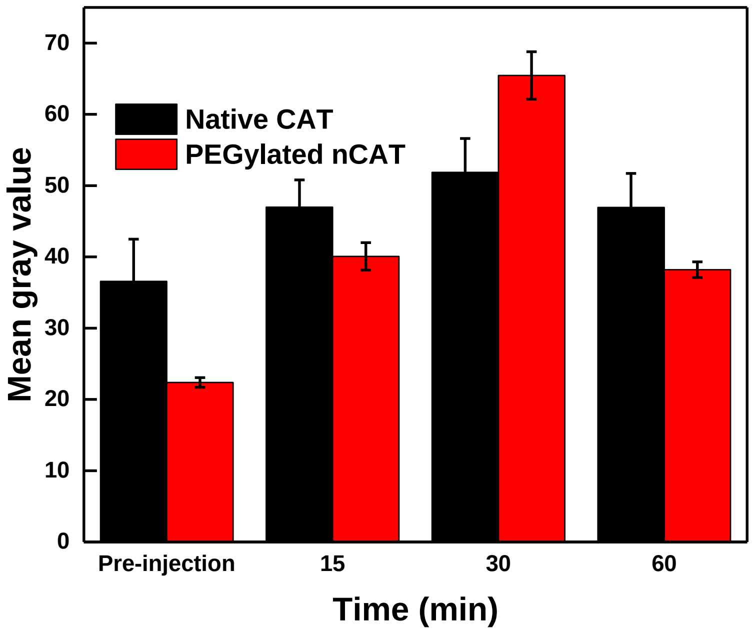

Figure S10. Mean gray values of the 4T1 tumor before and after intravenous injection of the native CAT and the PEGylated nCAT.

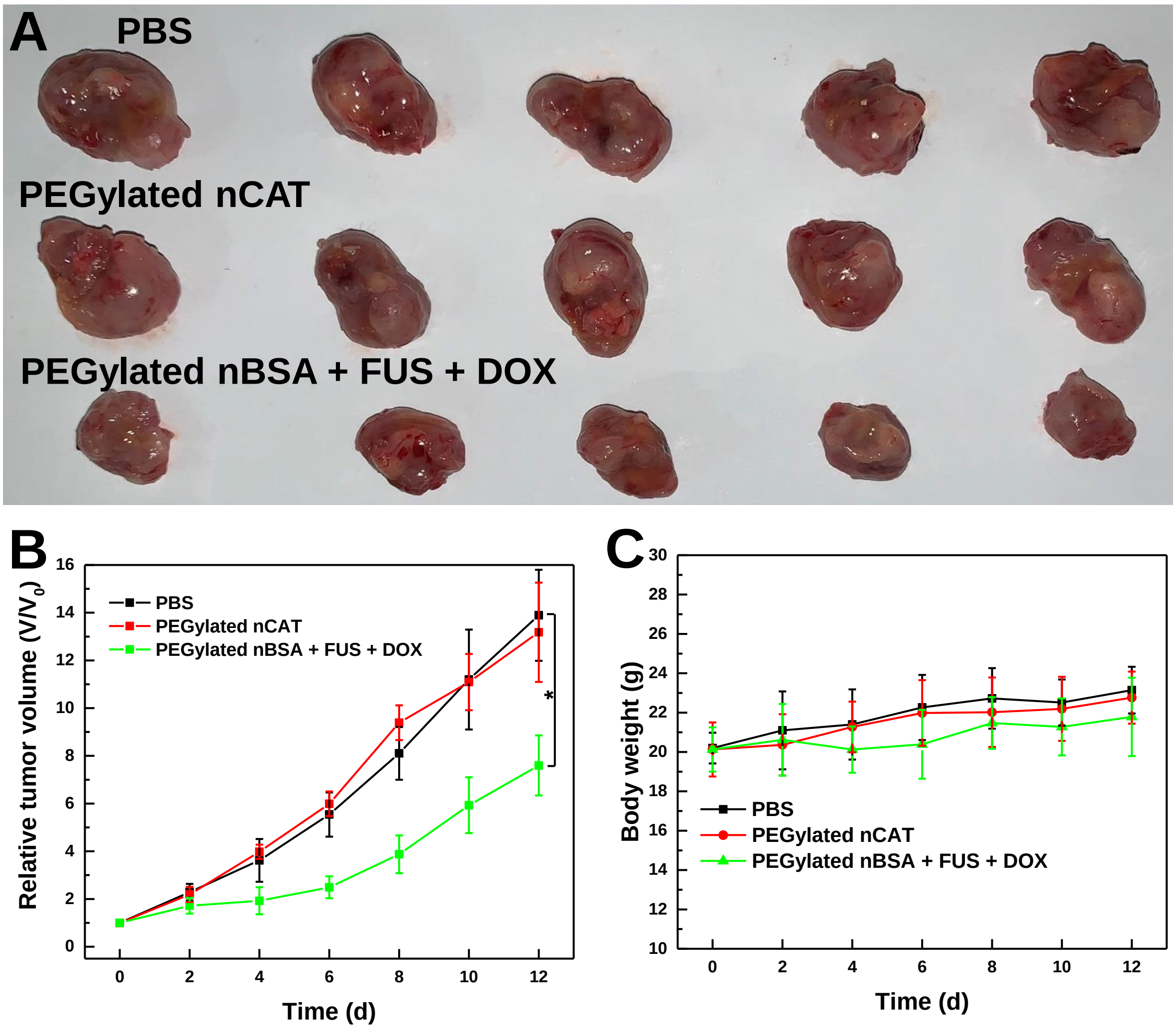

Figure S11. (A) Photograph of tumors after the in vivo therapy. The dosage of DOX and PEGylated nCAT/nBSA is 5 mg/kg and 10 mg/kg, respectively. FUS was conducted with the settings of 1.5 W/cm^2 and 50% duty circle for 3 min. (B) Tumor growth curves after the different treatments. Tumor volumes (V) were normalized to their initial values (V_0). (C) Mice body weight changes of different groups over 12 d.

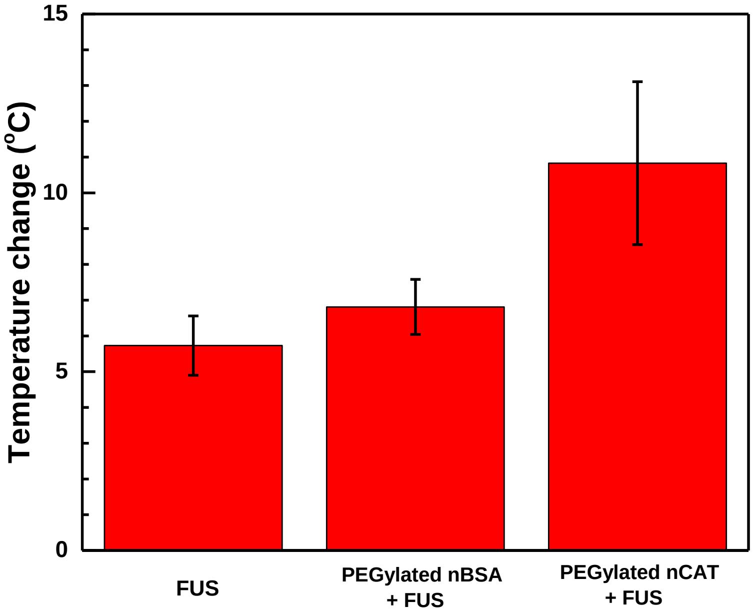

Figure 12. Tumor position temperature change pre- and post-FUS treatment after intratumoral injection of PBS, PEGylated nBSA and PEGylated nCAT. FUS was conducted with the settings of 1.5 W/cm^2 and 50% duty circle for 3 min.

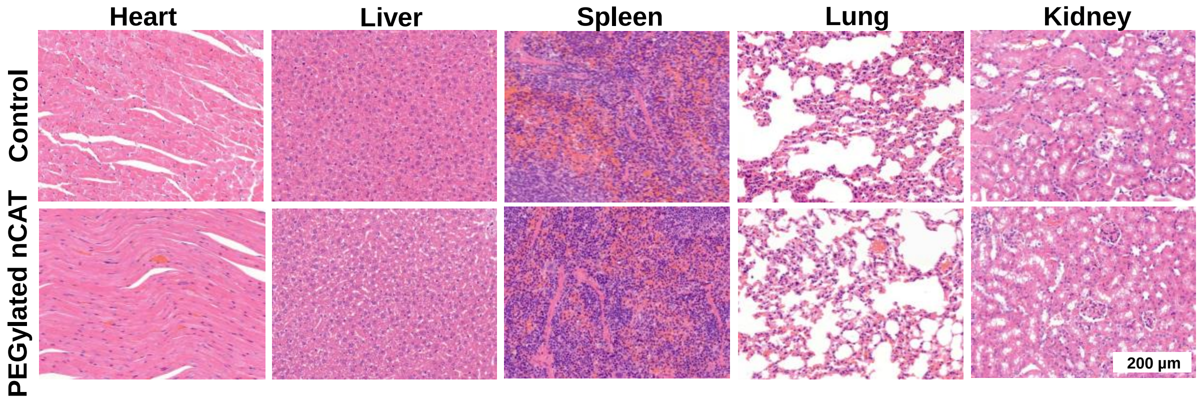

Figure S13. H&E staining of heart, liver, spleen, lung and kidney from healthy mice at 30 d post-injection of the PEGylated nCAT (200 μL, 1 mg/mL). Mice without any treatment were used as control.
